# On the Value of the Operation of Extraction of Cataract, and on the Use of Anœsthetic Agents in Ophthalmic Surgery

**Published:** 1851-07

**Authors:** 


*On the value of the operation of Extraction of Cataract, and on the use of Anæsthetic agents in Ophthalmic Surgery.* By Henry W. Williams, M. D., Boston.

Sir,—Whilst we endeavor, by careful observation and reflection, to contribute our share toward increasing the efficiency of the art of healing, it is no less important to maintain undiminished the resources with which it has been enriched by the labors of our predecessors.

This consideration prompts me to ask the opportunity of recalling the attention of the profession, through the pages of your Journal, to an operation which seems to have been appreciated among us (at least in New England) below its real value, and to have fallen, in some measure, into neglect and disuse.

I refer to the operation for the extraction of cataract, and particularly to that method of performing it which is effected through a section of the upper half of the cornea.

One of the reasons which have contributed to cause the operation to be regarded with disfavor, was the mistaken zeal which led some of its earlier partizans to perform it, indiscriminately, in all cases of cataract.

As we meet with several varieties of this affection, essentially differing in their physical characters, we may readily infer that a mode of operation which is best adapted for one form, may be very inapplicable to another.

A large number of cases, comprising the very soft and the fluid cataracts, are best disposed of by the process of division. It is only between the methods of displacement or couching, and by extraction, that comparison is to be made.

If the last-named operation is successful, it accomplishes a radical cure. We no longer have a foreign body in the eye, liable by its hard consistence or its increased volume to cause inflammation, or by its re-ascension to create a necessity for a second operation. Extraction has also claims to superiority from the more perfect degree of vision which ensues from its success.

The advantages I have mentioned, especially the complete exemption from any secondary accidents, have entitled this operation to be regarded, by the most distinguished surgeon oculists of Europe, as the method, *par excellence*, in the cases to which it is appropriate.

The cases to which it is peculiarly applicable are those of semi-hard cataract (which is one of the forms most commonly met with) as this variety sometimes rapidly increases in bulk after reclination, and in this swollen state is liable, by its pressure, to provoke serious, if not destructive inflammation of the internal tissues. It is especially desirable to employ this method in operating upon persons of advanced age, as, in such, the lens is not readily removed by absorption. Very hard cataracts are dissolved with difficulty at any period of life, and as they are apt, if merely displaced, to cause lesion or compression of the retina, the operation of extraction may be regarded as indicated. The same indication exists in cases where there is any tendency to amaurosis or to cerebral congestion, as the retina may then be injuriously affected by the slightest pressure from the displaced lens.

On the other hand, it is equally important to avoid attempting extraction under unfavorable circumstances; as when the eye is too small or too deeply sunken in the orbit to allow room for making a proper section of the cornea—

when the pupil is contracted, or adherent to the capsule — or when we have evidence that softening has taken place in the vitreous humor.

Oscillation of the globe was formerly considered as contra-indicating this mode of operating, but we have now the means of controling this unsteadiness by anæsthetic agents.

I do not know whether any other person has employed these auxiliaries in operations upon the eye. When in Europe, soon after the discovery of these adjuvants in surgical practice, the most distinguished authorities expressed to me their opinion that they should not be made use of in ophthalmic surgery, and I have never heard of their being thus applied by any other operator. My own experience, however, has proved, that we may safely avail ourselves, occasionally, of their aid, and thus obtain the opportunity to execute our manœuvres with more ease, and therefore with greater certainty.

Since my first resort to them, more than two years since, I have several times employed ether or chloroform in operations for cataract, and have thus secured complete success in cases of unusual difficulty.

A second reason for the prejudice which has prevailed against the extraction of cataract, is to be found in the formidable descriptions which have been given of the accidents that may follow this operation, and of the uncertainty attending it.

But these unfavorable statements of consequences and results have been mostly based upon operations in which the section of the cornea has been made at its inferior margin.

The section of the upper half is attended with a little more difficulty than that along its lower edge, but it offers very important advantages. The risk of prolapsus iridis, or of evacuation of the vitreous humor, either at the moment of the operation, or subsequently, is in a great measure avoided; and the flap of the cornea being covered by the upper eyelid, and the two edges of the wound thus kept in exact opposition, every opportunity is afforded for immediate union.

If, on the contrary, the incision is made downward, there is danger that the adhesion of the wound may be prevented by the secretions with which it is bathed in the lower palpebral sinus — by the constant trickling of the humors from the interior of the eye — or by a prolapse of the iris; or that the edge of the lower lid may insinuate itself beneath the flap, and, by everting it, cause the discharge of the entire contents of the globe.

It is not to be denied, that the operation by extraction, is slightly more difficult than those executed with the needle; but this will not justify us in consigning to oblivion one of the highest glories of surgical achievement.

The actual dangers attending its performance are not greater than those incident to other methods, and, its manœuvres once skillfully terminated, the patient is placed under conditions of far greater security against future accidents.

For all these reasons, this operation, without assuming any title to exclusive adaptation, deserves to be regarded as often the most advantageous, and sometimes the only mode on which we can rely; and claims, not only a place, but a high position, among the means by which our profession relieves "the ills that flesh is heir to."

The results of my own experience have confirmed the high opinion of the value of this method which I had derived from observation of the practice of the most distinguished European operators, in whose hands I was satisfied that in a very large number of cases a success was obtained much greater than would have followed operations with the needle upon the same subject.

10 Essex-st., Boston, June 6, 1851. *Boston Med. and Surg. Jour.*

